# Swallowing a "Precious Morsel"—A Plate and Eleven Teeth

**Published:** 1847-03

**Authors:** 


*Swallowing "a Precious Morsel"—a Plate and Eleven Teeth.*—We received a few days since from Mr. William. Dalrymple, dentist, of Homer, Courtland County, New York, an account of one of the most remarkable cases of *tooth swallowing* which has ever come to our knowledge. We have often heard of persons swallowing single teeth, but we had not conceived it possible to swallow nearly a whole set at once, plate and all. We should publish the report entire, did our space admit of it, but as we are unable to do this, we will present the main facts, which will doubtless both interest and astonish every reader of the Journal. The patient, Mr. H——, was a gentleman some 30 years of age.

He was wearing a plate of ***eleven*** teeth, which were fastened by means of several gold pivots, going into as many roots, which had been left in the jaw, which at the time the plate was set (about five years before) were sound. These roots, had gradually decayed, and the pivots become correspondingly loose, till it required a considerable care to keep the plate in place. "On the night of the 10th, of Dec." says the report, "Mr. H. awoke from his sleep with a sense of strangulation. He immediately arose from his bed, and made several attempts at swallowing—afterwards taking a draught of water. He immediately procured a light, and returned to the bed; by this time he discovered, that his plate was missing, and not finding it in the ***bed, which he had often done before,*** it occurred to him he had swallowed it!! The sense of strangulation, still continuing, though diminished in a degree, he immediately dressed himself, and proceeded to his physician!

The case being examined, and the nature of it ascertained, two other physicians were sent for. They finding that he had in fact swallowed his plate, and ascertaining its exact situation, proceeded to devise and prepare an instrument to remove it. This was finally effected, by forming a blunt hook from copper wire.

The report goes on to state, that "after the descent of the plate below the larynx, the patient did not suffer much from difficulty of breathing, for the position of the plate was such as to make the pressure mainly lateral, which tended to relieve the cavity of the trachea. Had the plate lodged in that part of the pharynx, contiguous to the epiglottis, it would inevitably have produced by its irritation, permanent spasm of the epiglottis, and consequently, sudden death. It did not give him pain, except when he attempted to swallow. The plate in its longest dimensions was full $2\frac{1}{8}$ inches, and was in the throat three hours. The patient was perfectly quiet, during the whole operation, with the exception of an occasional gagging." Mr. Dalrymple, closes his report by the following very just remark—"I hope this case, will be a caution to those who are in the *daily* habit of *pivoting plates.*"

*Syracuse Ed.*

